# Desiccation tolerance in streptophyte algae and the algae to land plant transition: evolution of LEA and MIP protein families within the Viridiplantae

**DOI:** 10.1093/jxb/eraa105

**Published:** 2020-02-28

**Authors:** Burkhard Becker, Xuehuan Feng, Yanbin Yin, Andreas Holzinger

**Affiliations:** 1 University of Cologne, Cologne, Germany; 2 University of Nebraska-Lincoln, Lincoln, NE, USA; 3 University of Innsbruck, Department of Botany, Innsbruck, Austria; 4 University of Osnabrück, Germany

**Keywords:** Charophyta, late embryogenesis abundant protein, LEA, major intrinsic protein, MIP, Streptophyta, terrestrialization, transcriptome, *Zygnema*

## Abstract

The present review summarizes the effects of desiccation in streptophyte green algae, as numerous experimental studies have been performed over the past decade particularly in the early branching streptophyte *Klebsormidium* sp. and the late branching *Zygnema circumcarinatum*. The latter genus gives its name to the Zygenmatophyceae, the sister group to land plants. For both organisms, transcriptomic investigations of desiccation stress are available, and illustrate a high variability in the stress response depending on the conditions and the strains used. However, overall, the responses of both organisms to desiccation stress are very similar to that of land plants. We highlight the evolution of two highly regulated protein families, the late embryogenesis abundant (LEA) proteins and the major intrinsic protein (MIP) family. Chlorophytes and streptophytes encode LEA4 and LEA5, while LEA2 have so far only been found in streptophyte algae, indicating an evolutionary origin in this group. Within the MIP family, a high transcriptomic regulation of a tonoplast intrinsic protein (TIP) has been found for the first time outside the embryophytes in *Z. circumcarinatum*. The MIP family became more complex on the way to terrestrialization but simplified afterwards. These observations suggest a key role for water transport proteins in desiccation tolerance of streptophytes.

## Teaser

Adaptations to environmental stress such as desiccation are needed for land plant transition. Late embryogenesis abundant (LEA) proteins and major intrinsic proteins (MIPs) diversified in ancestors of early land plants.

## Introduction

**Fig. 1.**
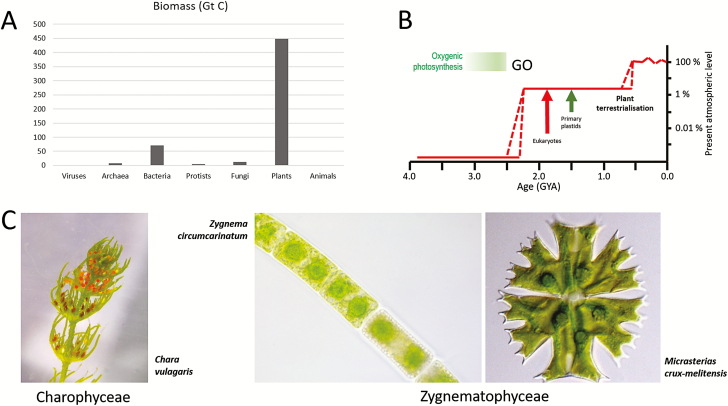
Importance of plant terrestrialization. (A) Biomass distribution on earth according to Bar-On *et al*. (2018. (B) Change in the atmospheric oxygen level during earth history. Reprinted by permission from Springer Nature. Lyons *et al*. (2014). The rise of oxygen in Earth’s early ocean and atmosphere. Nature **506,** 307–315. Copyright 2014. Broken lines: alternative scenarios; GO, great oxygenation event. Possible ages for eukaroytes, primary plastids, and plant terrestrialization are indicated. (C) *Chara vulgaris* a representative of the Charophycae, which has often been suggested to be the sister of land plants. *Zygnema circumcarinatum* and *Micrasterias crux-melitensis* represent the filament and unicell life forms of the Zygnematophyceae, the sister clade to land plants. Image of *Micrasterias crux-melitensis* courtesy of Dr Monika Engels, Hamburg; with permission.

The colonization of terrestrial habitats by plants was accompanied by exposure to a number of abiotic stress factors such as high irradiance, lack of mineral nutrients (bare rocks, no soils), varying temperature, and dehydration (Rensing, 2018). These stress factors required extensive adaptations at the molecular, cellular, and organismal level. The evolution of a heteromorphic life history (Becker and Marin, 2009) and symbiotic relationships with soil-borne fungi including arbuscular mycorrhizal (AM) fungi (Parniske, 2008) were two important innovations to cope with these stresses. Strikingly, the colonization of land by plants was so successful that land plants today represent 80% of the total biomass on earth (Fig. 1A; Bar-On *et al.*, 2018), even though land plants contribute only ~50% to the global primary production. Thus, while plant biomass in the marine environment shows a high turnover, plant biomass in the terrestrial habitat is much more stable and has accumulated after terrestrialization. The production of large amounts of plant biomass by aerobic photosynthesis led to the accumulation of oxygen in the atmosphere. Only after colonization of the land by plants did oxygen in the atmosphere reach levels required to sustain complex and large animals (Fig. 1B).

An additional major difference between aquatic and terrestrial primary production is that in the aquatic environment several different polyphyletic algal groups (e.g. diatoms, haptophytes, brown algae, red algae, green algae, and cyanobacteria) contribute to primary production. In contrast, on land, most of the primary production is from a single monophyletic group, the land plants (embryophytes). Therefore, plant terrestrialization was a unique and pivotal moment in the evolution of life on earth and, without it, our world would look completely different. While it has been clear for some time that embryophytes evolved from streptophyte algae (charophytes; Becker and Marin (2009) the exact sister group has only been recently determined (Fig. 1C). The morphological complex stoneworts [macrophytes (Fig. 1C) superficially resembling herbaceous plants] have been regarded for a long time as sister to land plants. However, recent work has now firmly established that the sister to land plants are the conjugating green algae (Zygnematophyceae; Fig. 1C; Wodniok *et al.*, 2011; Timme *et al.*, 2012; Wickett *et al.*, 2014; One Thousand Transcriptome Inititative, 2019).

Among the many adaptations of embryophytes, desiccation tolerance is of utmost importance for the colonization of the terrestrial habitat. Terrestrial organisms are exposed to dry air, and water availability varies during the day/night cycle and/or seasons of a year. The algal ancestors of modern embryophytes developed morphological, physiological, and molecular adaptations to survive this abiotic stress. In the following, we will first discuss what is known about survival strategies of (streptophyte) algae, when exposed experimentally to desiccation stress, and then discuss as an example the evolution of two protein families [late embryogenesis abundant (LEA) proteins and the major intrinsic protein (MIP) family] during plant terrestrialization.

## Desiccation tolerance in streptophyte algae

Removal of larger proportions of water from streptophyte algal cells represents a severe, in many cases lethal, stress. As the structure of biomolecules and membranes is maintained by water molecules, dehydration above a certain threshold leads to an irreversible aggregation of macromolecules and disintegration of organelles. In general, we can define desiccation tolerance as the ability to survive drying to ~10% remaining water content, being equivalent to ~50% relative air humidity (RH) at 20 °C (=water potential of –100 MPa) (Alpert, 2006; Oliver *et al.*, 2010). This physical limit prevents many groups of streptophytes from leaving the protecting water body. In the present review we will focus on streptophyte algae, even though it is known that within the chlorophyte lineage many freshwater members are also known to tolerate severe water loss (Gray *et al.*, 2007). We will not discuss the strategy of lichenization, which occurs almost exclusively in chlorophytes, and the drastically differing mechanisms of marine green macroalgae, also belonging to the chlorophytes. All these organisms have in common that they are poikilohydric and cannot actively regulate their water content, which can easily lead to desiccation under water-limited conditions. This is in contrast to homoiohydric land plants equipped with a cuticle, Casparian strip, and guard cells for active control of water loss and redistribution.

The effects of desiccation were recently tested in several members of the Klebsormidiales (Karsten *et al*., 2014, 2016) and Zygnematales (Pichrtová *et al.*, 2014a; Herburger *et al.*, 2015). In this research approach, a defined volume of algae was exposed to a pre-set relative humidity (RH) and the physiological reaction was investigated by measuring the effective quantum yield from the outside of the test chamber (Karsten *et al.*, 2014). This technique allows determination of which RH leads to a reduction of the effective quantum yield to zero, but does not allow measurement of the absolute water content of the desiccated cells. With this technique, several members of *Klebsormidium*, including the African strains from the G-clade and one member of the E-clade (Karsten *et al.*, 2016), and different members of *K. flaccidum* (Karsten *et al.*, 2017) were investigated. The G-clade member desiccated at this experimental setting between 300 min and 400 min, and a recovery was seen in three of the investigated strains, and the strain that did not recover was composed of single cells or small filaments (Karsten *et al.*, 2016a). General physiological effects of desiccation in green algae have been summarized previously (Holzinger and Karsten, 2013; Karsten and Holzinger, 2014).

A particularly interesting observation was made on the desiccation tolerance of *K. flaccidum* where the terrestrial strain (SAG 2307) maintained ~20% of the initial effective quantum yield even 22 h after onset of the experiment, recovered much better than an aquatic strain of *K. flaccidium* (SAG 7.91), and at 8 h after rehydration ~50% of the initial value was restored (Karsten *et al.*, 2017). In contrast, the aquatic strain of *K. flaccidium* at this time point showed only 20% recovery and, even after 50 h, only half of the initial value was reached. Lajos *et al.* (2016) determined a relative volume reduction at a given RH, illustrating that *Zygnema* and *Klebsormidium* could reduce their protoplasmic volume by ~80% of the initial value at the lowest RH of ~10%. This confirms earlier investigations by light microscopy and TEM, where a severe shrinkage was observed in *K. crenulatum* (Holzinger *et al.*, 2011). In general, the ability to aggregate may be very beneficial for avoiding water loss in *Klebsormidium* (Karsten and Holzinger, 2014; Karsten *et al.*, 2014). Another approach to remove water from algal cells is to incubate them in osmotically active substances, such as non-hydrolyzable sugars (Kaplan *et al.*, 2013, 2012; Pichrtová *et al.*, 2014b).

Pierangelini *et al.* (2019) stated that light and dehydration drive adaptations of early branching streptophytes *Hormidiella*, and the newly described genera *Streptosarcina* and *Streptofilum*. Two isolates of *Streptosarcina arenaria* (SAG 2560 and SAG 2562) desiccated within ~400 min and showed recovery to ~80% of the initial value within 100 min (Pierangelini *et al.*, 2019). Both strains are characterized by the formation of cell packets and a low light adaptation. In contrast, *Streptosarcina costaricana*, the newly described genus *Streptofilum capillatum*, and *Hormidiella parvula* desiccated much faster in ~200 min, and did not recover the effective quantum yield after rehydration (Pierangelini *et al.*, 2019). The relevance of cell shape for desiccation tolerance is also found in an advanced member of the streptophytes. Submersed *Coleochaete* usually form ﬂat epiphytic disks or cushion-like thalli, composed of densely branched ﬁlaments. In contrast, when grown in aero-terrestrial conditions (on agar or sand) it markedly changes its morphology and growth habitus. It forms multistratose clusters of thick-walled cells with acetolysis-resistant autoﬂuorescent cell wall components (Graham *et al.*, 2012).

**Fig. 2.**
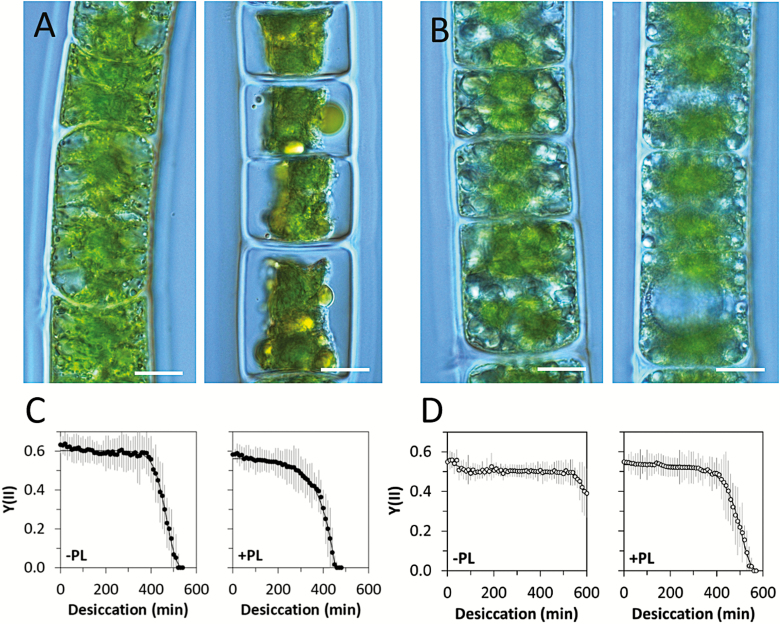
Effects of desiccation in *Z. circumcarinatum*. (A) Young filaments (1 month); young filament desiccated at 86% RH for 2 h (right), (B) old filaments (1 year); old filament desiccated at 86% RH for 2 h (right), scale bars=10 µm, (C) young filaments desiccated without or with pectate lyase (–/+PL), (D) old filaments desiccated without or with pectate lyase (–/+PL). Note the different time spans needed until the effective quantum yield dropped to zero. (C and D) are after Herburger *et al.* (2019).

While poikilohydric plants cannot actively control their water content, it has long been speculated about possible functions of, for example, pectic components in water-holding capacity and thus an increase in desiccation tolerance. Young, 1-month-old *Zygnema circumcarinatum* cells are visibly more greatly damaged by desiccation at 86% RH (Fig. 2A) than 1-year-old cells (Fig. 2B). Herburger *et al.* (2019) investigated in detail the effects of the pectic component homogalacturonan on desiccation tolerance. While young, ~1-month-old, cultures of *Z. circumcarinatum* desiccated ~500 min after onset of the experiment, cells treated with pectate lyase took only ~450 min to desiccate (Fig. 2C). When cells were treated with pectate lyase and then allowed to recover for 12 h, the full initial protection of the pectic layer was achieved (Herburger *et al.*, 2019). In contrast, old filaments (Fig. 2D) were very well protected and did not desiccate by 600 min, but this effect was reduced by the application of pectate lyase (Fig. 2D). It has been demonstrated several times and in different members of the genus *Zygnema* that older cultures are much better protected against desiccation (Pichrtová *et al.*, 2014a; Herburger *et al.*, 2015). A correlation with a more prominent pectic layer was found by Herburger *et al.* (2019), where 12-month-old cultures did not desiccate up to 600 min. In contrast, the occurrence of arabinogalactan proteins in the outer layer of the cell wall in streptophytes has mostly been held responsible for adhesion phenomena (Palacio-López *et al.*, 2019). A different strategy to withstand desiccation is realized in *Klebsormdium* cell walls (Holzinger *et al.*, 2011; Herburger and Holzinger, 2015), where the cell walls are rich in callose, which gives them a high degree of flexibility, beneficial to avoid mechanical damage upon desiccation. We can summarize that aggregation of cells, ﬂexible cell walls, mucilage production, and accumulation of osmotically active compounds are the most common desiccation tolerance strategies in streptophytes.

## Transcriptomics of desiccation tolerance in streptophyte algae

**Fig. 3.**
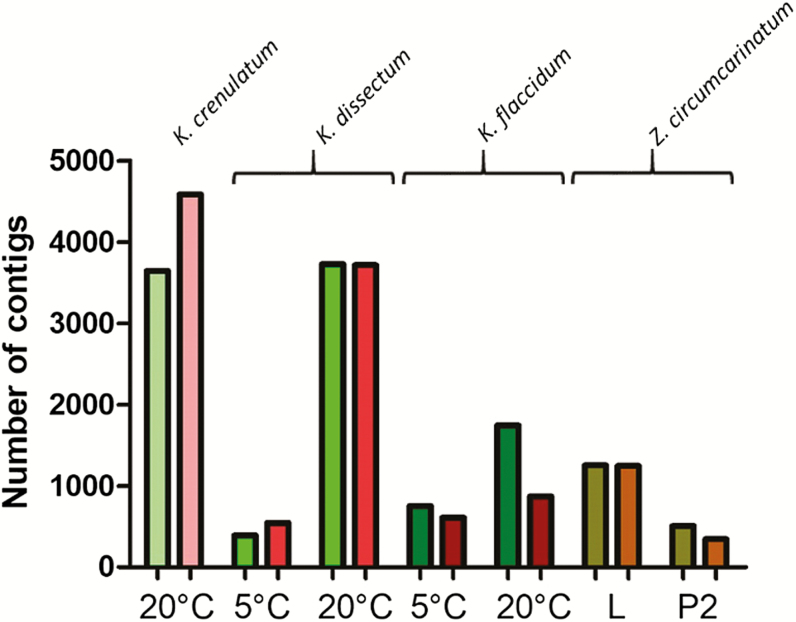
Overall changes of gene expression levels in different *Klebsormidium* species and *Zygnema circumcarinatum*. Left (greenish) bars: number of up-regulated transcripts. Right (reddish) bars: number of down-regulated transcripts. The *Klebsormidium* experiments were performed at either 5 °C or 20 °C (data from Holzinger *et al.*, 2014; Rippin *et al.*, 2019a). For *Z. circumcarinatum*, the type of cultivation (L, liquid culture, 1 month old; P2 agar plate culture, 7 months old) is indicated (data from Rippin *et al.*, 2017). The temperature in the *Z. circumcarinatum* experiment was 20 °C.

Recently, we have started to investigate the molecular response to desiccation stress in laboratory experiments. Our work has centered on different species of the early branching streptophyte *Klebsormidium* (Holzinger *et al.*, 2014; Holzinger and Becker, 2015; Rippin *et al.*, 2019a) and the late branching *Z. circumcarinatum* (Rippin *et al.*, 2017) which gives its name to the genus of the Zygnematophyceae, the sister group to land plants. Others have investigated the transcriptional changes upon stress response in *Z. circumcarinatum* to different abiotic stressors [osmotic stress (Fitzek *et al.* 2019); temperature and light (de Vries *et al.* (2018); and light *in situ* (Rippin *et al.* (2019b)]. In the present review article, we will concentrate on our desiccation stress experiments; however, it should be noted that there is generally considerable overlap between the cellular response to abiotic stressors such as temperature (cold), osmotic, and desiccation stress. The overall change in expression levels of the transcripts upon desiccation stress in our experiments is highly variable (Fig. 3). The variability is partially due to differences in the experimental set-up (liquid culture or agar plate culture as starting material, different temperatures). For example, 1-month-old liquid cultured *Z. circumcarinatum* cells show a much stronger response compared with 7-month-old agar plate cultured material (Rippin *et al.*, 2017). Furthermore, there is only a small overlap between both conditions: 317 (140) transcripts are up- (down-) regulated, respectively. However, even under the same experimental conditions using the same bioinformatic analysis parameters, different species can have remarkably different qualitative and quantitative gene expression responses (compare *K. dissectum* with *K. flaccidum* in Fig. 3 and see fig. 8 in Rippin *et al.*, 2019a for differences in the gene repertoire regulated). These analyses have led to large data sets that allow comparison of the desiccation stress response of streptophyte algae to embryophytes.

However, before we go into details, two problems associated with this type of study should be briefly discussed. First, the number of transcripts that can be annotated is, for many of these non-model species, still low. For example, in the case of the *Z. circumcarinatum* stress transcriptome, >60% of the up- and down-regulated transcripts cannot be assigned any function. Secondly, while the established transcriptomics gives interesting information about the changes of the transcript levels for many genes, these transcripts are not directly responsible for the cellular response. The translation efficiency of mRNAs and the protein degradation rate can vary considerably for different proteins and different experimental conditions, and, therefore, modify the gene expression response (reviewed in Liu *et al.*, 2016). In addition, protein activity can be post-translationally regulated. For this reason, a change in mRNA level might not translate into a measurable physiological or biochemical effect at all, and it would be important to combine transcriptomics with proteomics and metabolomics for a complete understanding of a cellular response to abiotic stress. Generally speaking, post-transcriptional events regulate fast responses, while differences in the transcriptome level are very stable once a new steady state has been reached (Liu *et al.*, 2016).

Overall the response to desiccation stress in *Klebsormidium* and *Zygnema* is very similar to the response of land plants to the same stressor, as already stated in the title of the first *Klebsormidium* publication on transcriptomic changes (Holzinger *et al.*, 2014). Any plant cell must protect the cellular structure (membranes) including the photosynthetic apparatus from the damaging direct effects of protein denaturation caused by the water loss of the cell upon desiccation. The inactivation of proteins due to water loss is responsible for the cessation of photosynthesis, leading to damaging effects of radiation [direct or indirect due to the production of reactive oxygen species (ROS) created by the absorption of light]. However, it is still surprising that nature apparently relied on only a small number of proteins and processes to protect the cells under these dangerous conditions. Chaperones are up-regulated and osmolytes accumulate to protect the cellular structure. The chaperones that protect the photosynthetic apparatus generally include ELIPs (early light-induced proteins), while other structures are protected by several different chaperones including LEA proteins, and both of these protein families have members that are up-regulated in streptophyte algae upon desiccation stress. The accumulating osmolytes in algae include sugars, amino acids, and sugar alcohols, all commonly used in land plants too. In *Klebsormidium*, we even observed the up-regulation of the RFO (raffinose family oligosaccharides) pathways typically involved in cold, osmotic, and desiccation stress response in plants. To protect the cells from ROS, we observed up-regulation of glutathione- and ascorbate-related pathways, again typical responses to desiccation and other abiotic stresses in plants. Finally, some phytohormones known to play a role in abiotic stress response in embryophytes [abscisic acid (ABA), ethylene, and cytokines] might also play a role in abiotic stress response, at least in *Klebsormidium* (Holzinger and Becker, 2015).

Are there any differences in the desiccation response between streptophyte algae and embryophytes? While the basic concepts are identical in both groups of organisms, there are some major differences. In the streptophyte algae investigated, the desiccation response is mainly a response of individual cells to the stressor (although we cannot exclude completely that cells communicate with each other by, for example, ABA; see above). In contrast, in embryophytes, all organisms are multicellular, and cell differentiation and the response of the individual cells must be a coordinated response of the organism, not the individual cell, in order to achieve survival. Therefore, it is not surprising that embryophytes use a much more elaborate communication network, which includes new phytohormones (jasmonic acid, salicylic acid, etc.) and an expansion of the number of members within a phytohormone network; for example, the PYR/PYL receptor for ABA has expanded to 14 isoforms in *Arabidopsis*. This is a general pattern observed in the evolution of land plants: Upon terrestrialization, the number of subfamilies within a protein family and the number of the members of the protein family increased in embryophytes when compared with streptophyte algae. In the final part of this review, we will present this in more detail for two protein families important for survival upon desiccation: the LEA protein and MIP families.

## The LEA protein superfamily

LEA proteins were initially discovered accumulating late in embryogenesis in plant seeds (Hundertmark and Hincha, 2008). LEA proteins protect other proteins from aggregation due to desiccation, osmotic stresses, and low temperature (Goyal *et al.*, 2005; Shinde *et al.*, 2012; Hatanaka *et al.*, 2013) using mechanisms which are distinct from those displayed by heat shock molecular chaperones. LEA proteins have been grouped into nine different subgroups (Table 1) based on their sequence motifs/patterns (Hundertmark and Hincha, 2008). Drought/desiccation-induced expression of LEA proteins has already been reported for several species. An interesting aspect is the distribution of these chaperones across the Viridiplantae (Table 1; see Rippin *et al.*, 2017 for details). Chlorophyte and streptophyte algae encode LEA4 and LEA5 proteins (Table 1), suggesting that these two groups probably emerged early and were already present in the last common ancestor of Viridiplantae. All analyzed streptophyte genomes encode LEA2 proteins that so far have not been found in chlorophyte algae, indicating an origin after the split of chlorophytes from streptophytes. Another interesting member of the LEA proteins is dehydrin, which was only reported for *Dunaliella salina* within the green algae. Whether the presence of dehydrin in *Dunaliella* is due to a late acquisition or an ancient state with subsequent loss in most green algal lines needs to be examined in more detail. SMP (seed maturation protein) is absent from all published algal genomes. All other LEA subfamilies [PvLEA18 (*Phaseolus vulgaris* LEA18), LEA1, LEA3, and ATM (named ATM for unclear reasons by Raynal *et al.*, 1999)] probably evolved within tracheophytes as they are absent from bryophytes (Table 1). In the context of plant terrestrialization, LEA proteins originating in streptophyte algae, therefore, might have provided a basal protection during desiccation stress. The last ancestor of bryophytes and and tracheopyhtes might have added two additional subfamilies for better protection. Thus, these proteins are prime targets for the study of the evolution of desiccation response during terrestrialization. However, not all LEA proteins may be involved in stress response. Some might serve other functions. For example, in the *K. crenulatum* stress transcriptome (Holzinger *et al.*, 2014), 12 LEA proteins were found. Most of them are up-regulated upon desiccation stress, while two are down-regulated and one is not regulated at all during this stress.

**Table 1.** Distribution of late embryogenesis abundant (LEA) proteins within the green plants

Protein	*A. thaliana*	*P. patens*	*M. polymorpha*	Streptophyte algae	Chlorophyte algae
**SMP**	**+**	**+**	**+**		
**PvLEA18**	**+**				
**LEA5**	**+**	**+**	**+**	**+**	**+**
**LEA4**	**+**	**+**	**+**	**+**	**+**
**LEA3**	**+**				
**LEA2**	**+**	**+**	**+**	**+**	
**LEA1**	**+**				
**Dehydrin**	**+**	**+**	**+**		**+**
**ATM**	**+**				

Grouping of LEA proteins is according to Hundertmark and Hincha (2008). Modified after fig. 1 in (Rippin *et al.*, 2017). In most organisms, each subfamily is represented by more than one member. *Arabidopsis* has at least two members in each subfamily and a total of 51 LEA proteins. For simplicity, all information for streptophyte and chlorophyte algae has been condensed and is presented in a single column for each of the groups. SMP, seed maturation protein

## The MIP superfamily

**Fig. 4.**
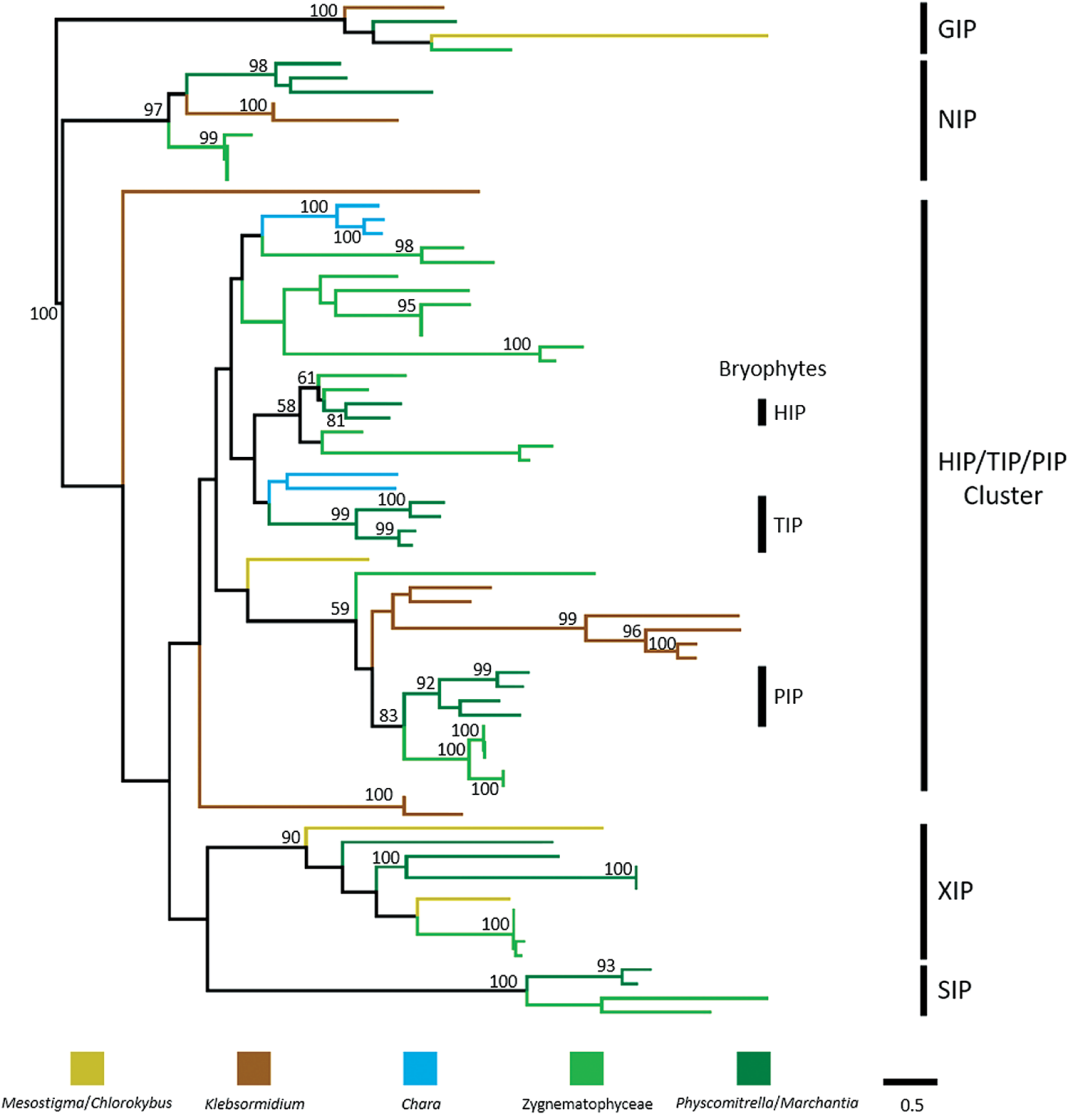
Phylogeny of MIPs within streptophytes: 78 protein sequences obtained from seven completed streptophyte genomes were analyzed using the phylogenetic pipeline by ETE3 (www.genome.jp/tools/ete/). See Supplementary Table S1 for a complete list of all proteins aligned. Alignment was performed using Clustal Omega v1.2.1 with the default options (Sievers and Higgins, 2014). The resulting alignment was cleaned using the gappyout algorithm of trimAI v1.4.rev6 (Capella-Gutiérez *et al.*, 2009). The best protein model was selected using NJ tree inference among LG, WAG, JTT, MtREV, Dayhoff, DCMut, RtREV, CpREV, VT, Blosum62, MtMam, MtArt, HIVw, and HIVb models using pmodeltest v1.4. An ML tree was inferred using RAxML v8.1.20 run with model PROTGAMMALGF and default parameters (Stamatakis, 2014). Branch supports were computed out of 100 bootstrapped trees. Boostraps ≥90 are indicated. In addition, a few bootstraps ≥50 within the HIP/TIP/PIP cluster are indicated.

The MIP family is an ancient protein family of channel (solute transport facilitators) proteins also named aquaporins. Most MIPs transport water but some also transport other solutes such glycerol, urea, and metalloids (e.g. arsenic acid, boric acid, and silicic acid). Aquaporins have been described, for example in the primitive vascular resurrection plant *Selaginella*, to be involved in osmotic adjustment leading to desiccation tolerance (Gu *et al.*, 2019). However, in the leguminous *Galega*, PIP aquaporins have been described to be involved in drought stress tolerance (Li *et al.*, 2015). Interestingly, two transcripts related to the HIP/TIP/PIP subfamilies are highly up-regulated (17-fold to 10 000-fold) upon desiccation in *Z. circumcarinatum* (Rippin *et al.*, 2017). Therefore, we decided to look into the evolution of the MIP family in green plants for this review (Fig. 4).

MIPs are found in most living organisms (humans have 13 known aquaporins; Day *et al.*, 2014). In plants, the protein family has expanded (*A. thaliana*: 35 known MIPs; Johanson *et al.*, 2001). Historically the plant MIP family has been divided into four subfamilies (Johanson *et al.*, 2001): PIPs (plasma membrane intrinsic proteins), TIPs (tonoplast intrinsic proteins), SIPs (small basic intrinsic proteins, in the endoplasmic reticulum), and NIPs (Nodulin-like intrinsic proteins). PIPs, TIPs, and SIPs are generally involved in water transport, whereas NIPs often seem to transport metalloids.  All MIPs have a very typical structure with six TMDs (transmembrane domains) and two loops dipping from the cytoplasmic and extracellular site into the membrane and creating the filter conferring specificity to the channel (Johanson *et al.*, 2001). Nearly all water channels have an NPA (asparagine–proline–alanine) amino acid motif within each loop, thought to be required for water specificity.

Danielson and Johanson (2008) examined the number and types of MIPs in *Physcomitrella patens*. Their work led to the recognition of three additional plant MIP subfamilies: GIPs (GlpF-like intrinsic proteins) which are generally glycerol transporters; XIPs (X intrinsic proteins) of unknown function; and HIPs (hybrid intrinsic proteins) of unknown function. Later work confirmed these subfamilies to be mainly absent in algal genomes, and green algal MIPs to be present in additional subfamilies called MIP A–E (Anderberg *et al.*, 2011). However, in 2011, this work could only cover the small genomes of a few prasinophytes as well as the few chlorophycean and trebouxiophycean genera sequenced at that time. A few years later, an extensive phylogenetic analysis of the diversity and evolution of MIPs was published by Abascal *et al.* (2014). Their work confirmed the seven embryophyte subfamilies and drew the following conclusions relevant to plant evolution: an ancient deep branching into glycerol transporters (termed the glp clade) and aquaporins (termed the aqp clade) (Abascal *et al.*, 2014). In plants, the glp clade is only represented by GIP proteins. All other plant MIP subfamilies belong to the aqp clade. Two other findings are also important: (i) the presence of XIPs outside plants in unicellular eukaryotes, suggesting an ancient origin with subsequent losses in many lineages; and (ii) the origin of SIPs in the last common ancestor of all green plants. However, at the time of publication of this important paper, no genomes from streptophyte algae had been reported and the number of chlorophyte genomes was still low. For our analyses, we searched streptophyte algal genomes (Hori *et al.*, 2014; Nishiyama *et al.*, 2018; Cheng *et al.*, 2019; Wang *et al.*, 2019), the *Z. circumcarinatum* draft genome, and moss genomes (Rensing *et al.*, 2008; Bowman *et al.*, 2017). Accession numbers for all protein sequences used for the analysis including a tentative assignment are presented in Supplementary Table S1 at *JXB* online. A detailed phylogenetic analysis is still ongoing, but for this review we have analyzed the MIP phylogeny in green plants using the phylogenetic analysis pipeline by ETE3 (https://www.genome.jp/tools-bin/ete). The resulting phylogenetic tree is shown in Fig. 4. GIPs, NIPs, SIPs, and XIPs are clearly present in various streptophyte algae, suggesting early evolutionary origins for these proteins in agreement with the findings of Abascal *et al.* (2014). However, several algal lineages apparently lack these proteins, suggesting line-specific losses. However, it is noteworthy that published transcriptomes of *Mesostigma* and *Zygnema* do contain a clear SIP ortholog, which has not been found in the genomes so far. Either there are cell line-specific differences, or the genomes are not complete with respect to the MIP complement. The large majority of streptophyte algal MIPs belong to the HIP/TIP/PIP clade. Unfortunately, different tree-building programs give different topologies, which in addition are not well supported by statistics. Therefore, it is very difficult to identify true orthologs to the HIP,   TIP, and PIP subfamilies in streptophyte algae. We currently cannot decide when TIPs evolved. The observed phylogenies (e.g. Fig. 4) indicate that TIPs probably evolved from HIP subfamilies, but whether the algal proteins are HIPs or true TIPs remains elusive. Blast analyses indicate the presence of   TIP-like proteins in many algae (even among chloropyhtes) but, with the exception of proteins from *Chara braunii* and *Z. circumcarinatum*, they never branch with TIPs in our phylogenetic analyses. Additional genomes from streptophyte algae will become available soon and might allow us to solve this puzzle. Why HIPs, XIPs, and GIPs were lost during land plant evolution is completely unclear.

## Concluding remarks

To summarize, streptophyte algae use the same basic mechanisms to achieve desiccation tolerance as poikilohydric land plants, suggesting that the algal ancestor of land plants was well prepared for this type of stress when it colonized the terrestrial habitat. In contrast, homoiohydric land plants developed different strategies such as the formation of a cuticle, Casparian strip, or guard cells to actively control their water content. Moreover, they expanded their molecular tool kit to counteract water loss as well as the communication network coordinating the organismal response. Both are probably required for the more complex (number of cell types) land plants to avoid water stress.

## Supplementary data

Supplementary data are available at *JXB* online.

**Table S1.** List of proteins used for phylogenetic analysis of the MIP family.

eraa105_suppl_Supplementary_Table_S1
