# Supplementary material for: Desiccation tolerance in streptophyte algae and the algae to land plant transition: evolution of LEA and MIP protein families within the Viridiplantae
Source: J Exp Bot. 2020 Feb 28;71(11):3270–8. doi: 10.1093/jxb/eraa105 (PMC7289719; doi:10.1093/jxb/eraa105)
Supplement: eraa105_suppl_Supplementary_Table_S1 [file eraa105_suppl_supplementary_table_s1.pdf]

Supplemental Table 1: Systematics, tentative assignment to subfamilies, no of amino acids and accession number of the protein sequences used for phylogenetic analyses (Figure 4) of the MIP protein family in streptophytes. *Physcomitrella patens* proteins were assigned according to Danielson and Johanson (2008). Proteins monophyletic with the *Physcomitrella patens* MIP subfamily with more than 90% bootstrap support were assigned to the same subfamily. Proteins monophyletic with less than 90% bootstrap support as subfamily like. A ? indicates proteins that could not be assigned to a specific subfamily.

Class	Species	Tentative assignment*	No of amino acids	Accession NO
Bryopsida	*Physcomitrella patens*	PIP1.1	289	XP_024369405.1
		PIP2.4	280	XP_024390253.1
		SIP1.1	277	XP_024403268.1
		TIP6.3	251	XP_024364314.1
		TIP6.4	251	XP_024382122.1
		SIP1.2	279	XP_024359131.1
		NIP1	246	XP_024359109.1
		XIP1	202	XP_024389628.1
		XIP2	201	XP_024385941.1
		NIP5.1	228	XP_024372976.1
		GIP	370	XP_024396316.1
		HIP	246	XP_024382299.1
Marchantiopsida	*Marchantia polymorpha*	XIP	326	PTQ48994.1
		XIP	320	PTQ48995.1
		TIP	253	PTQ50461.1
		TIP	252	PTQ29777.1
		HIP	421	PTQ28939.1
		PIP	327	PTQ27396.1
		PIP	307	PTQ39551.1
		NIP	302	PTQ47330.1
Zygnematophycea	*Spirogloea musicola*	PIP-like	286	SM000021S06406
		SIP	250	SM000093S24434
		PIP-like	286	SM000037S13523
		?	487	SM000090S24350

		NIP	366	SM000354S13406
		?	262	SM000200S05842
		PIP-like	290	SM000298S10941
		PIP-like	230	SM000050S17069
		PIP-like	252	SM000007S20799
		HIP/TIP/PIP Cluster	193	SM000022S07242
		HIP/TIP/PIP Cluster	322	SM000017S02872
		NIP	344	SM000117S25509
		NIP	344	SM000055S18272
		NIP	367	SM000318S12225
		HIP/TIP/PIP Cluster	136	SM004309S15688
		HIP/TIP/PIP Cluster	136	SM004508S16472
		HIP/TIP/PIP Cluster	108	SM004806S16624
	Mesotaenium endlicherianum	HIP	279	ME000025S04443
		PIP-like	367	ME000894S10208
		SIP	239	ME000329S05576
	Zygnema circumcarinatum	GIP	386	MT010839
		HIP/TIP/PIP Cluster	294	MT010840
		HIP/TIP/PIP Cluster	294	MT010841
		NIP	209	MT010842
		HIP/TIP/PIP Cluster	268	MT010844
		HIP/TIP/PIP Cluster	276	MT010845
		NIP	272	MT010846

		?	209	MT010847
		NIP	440	MT010848
		HIP-like	450	MT010849
Mesostigmatophyceae	*Chlorokybus atmophyticus*	NIP	345	Chrsp6S07497
		PIP-like	322	Chrsp35S05281
		GIP	488	Chrsp1S03082
	Mesostigma viride	XIP	359	Mesvi8S08640
Klebsormidiophyceae	*Klebsormidium nitens*	HIP-like	259	GAQ81723.1
		?	317	GAQ81008.1
		PIP-like	331	GAQ80027.1
		PIP-like	210	GAQ85890.1
		PIP-like	256	GAQ80531.1
		?	266	GAQ81006.1
		NIP	279	GAQ78837.1
		?	220	GAQ80844.1
		NIP	211	GAQ82426.1
		PIP-like	313	GAQ90274.1
		PIP-like	255	GAQ93600.1
		PIP-like	181	GAQ92437.1
		GIP	386	GAQ77677.1
Charophycae	*Chara braunii*	TIP-like	264	GBG70937.1
		HIP/TIP/PIP Cluster	289	GBG61162.1
		HIP/TIP/PIP Cluster	282	GBG61164.1
		HIP/TIP/PIP Cluster	290	GBG61166.1
		TIP-like	330	GBG92319.1
